# Two-Step Optimization for Spatial Accessibility Improvement: A Case Study of Health Care Planning in Rural China

**DOI:** 10.1155/2017/2094654

**Published:** 2017-04-18

**Authors:** Jing Luo, Lingling Tian, Lei Luo, Hong Yi, Fahui Wang

**Affiliations:** ^1^Hubei Provincial Key Laboratory for Geographical Process Analysis and Simulation, College of Urban and Environmental Science, Central China Normal University, Wuhan, Hubei 430079, China; ^2^Department of Geography & Anthropology, Louisiana State University, Baton Rouge, LA 70808, USA; ^3^Hubei Province Development Planning & Research Institute Co., LTD, Wuhan, Hubei 430071, China; ^4^Department of Real Estate, East China Normal University, Shanghai 200241, China

## Abstract

A recent advancement in location-allocation modeling formulates a two-step approach to a new problem of minimizing disparity of spatial accessibility. Our field work in a health care planning project in a rural county in China indicated that residents valued distance or travel time from the nearest hospital foremost and then considered quality of care including less waiting time as a secondary desirability. Based on the case study, this paper further clarifies the sequential decision-making approach, termed “two-step optimization for spatial accessibility improvement (2SO4SAI).” The first step is to find the best locations to site new facilities by emphasizing accessibility as proximity to the nearest facilities with several alternative objectives under consideration. The second step adjusts the capacities of facilities for minimal inequality in accessibility, where the measure of accessibility accounts for the match ratio of supply and demand and complex spatial interaction between them. The case study illustrates how the two-step optimization method improves both aspects of spatial accessibility for health care access in rural China.

## 1. Introduction

Location-allocation analysis seeks the optimal placement of facilities for a particular objective under various constraints. As outlined by Church [1], there are several classic location-allocation problems: the *p-median problem* minimizes the weighted sum of distances between users and facilities, the* location set covering problem (LSCP)* minimizes the number of facilities needed to cover all demand, and the* maximum covering location problem (MCLP)* maximizes the demand covered within a desired distance or time threshold by locating a given number of facilities, among others. Another popular model is the* minimax problem* with an objective of minimizing the maximum distance between users and facilities [2]. Most of the studies following this line of work emphasize efficiency, such as the *p*-median problem striving for cost saving in total travel distance, the LSCP attempting to cap the total commitment of resource, and the MCLP intending to max out the benefit of a given resource. Only the minimax problem marginally addresses the issue of equity as it minimizes the distance for the most remote user. Social scientists have long argued the dual and usually competing goals of efficiency and equity (e.g., [3–5]). In the rich body of literature on location-allocation analysis, the paucity of studies on modeling equity is evident. This is an area that merits more work especially when it comes to planning for public resources or services.

There are various principles of equity. For example, in health care, equity may be defined as equal access to health care, equal utilization of health care service, or equal health outcomes among others [6], and most agree that equal access is the most appropriate principle of equity from a public policy perspective [7]. This research emphasizes* spatial accessibility*, which refers to the convenience for residents at a given location to overcome the spatial impedance to obtain a service provided at a facility. Over the years, spatial accessibility measures have evolved from an emphasis on “proximity to supply locations” to accounting for complex interaction between supply and demand ([8]: 94). An early and simply measure of accessibility is minimum distance or travel time to the closest facility, which remains popular in the literature (e.g., [9, 10]). However, this approach assumes that residents only use the closest facility, and the capacity of facility is unlimited. One may use cumulative opportunities within a distance range [11] or a gravity-based potential model to add up distance-discounted opportunities [12] to account for multiple facilities valued by residents. When the scarcity of a service is a concern, recent advancement in accessibility measure, namely, the “2-step floating catchment area (2SFCA)” method [13] or its generalized version [14], considers capacities of facilities, demands of users, travel cost between them, and their match ratio in complex spatial interaction. In practice, residents usually value both proximity to the nearest facility and the availability of a service in terms of accessibility, perhaps with an order of priority. The methods such as the proximity measure and the 2SFCA capture different aspects of accessibility. The desire to account for both travel distance reduction and a reasonable ratio in supply capacity versus demand level can be also formulated explicitly in a planning problem. For example, Delmelle et al. [15] developed a Vintage Flexible Capacitated Location Problem (ViFCLP) to allow some assignments of demands to nonclosest facilities in order to gradually accommodate increasing demands by facilities with limited capacities.

There have been some recent studies modeling equal accessibility in location-allocation analysis. Based on the aforementioned 2SFCA-based accessibility index, Wang and Tang [16] introduced a new objective for planning facilities toward equal accessibility, defined as minimum inequality of accessibility for users across geographic areas. They used a quadratic programming (QP) approach to solve the model [16]. Similarly, Tao et al. [17] applied the model to planning senior residential facilities in Beijing and used a particle swarm optimization (PSO) method to solve the model. The decision option in both studies was to adjust the capacities of facilities to minimize the deviation of accessibility indexes. Wang et al. [18] simulated the decision options of adjusting capacities of existing facilities or siting new facilities in analyzing equal accessibility of National Cancer Institute hospitals and compared the outcomes in the two scenarios. Lately, Li et al. [19] proposed a two-step method to integrate location optimization and capacity optimization to fulfill the single objective of equal accessibility. They argued that a planning problem often required sequential decisions such as selecting the sites for new facilities first and then adjusting their capacities. However, their work fell short as it was on a simulated problem and calls for real world case study to support the rationale of the two-step decision process. Its singular focus on maximizing equality in both steps may not be completely acceptable to most planners, who also value other objectives related to optimal efficiency. More importantly, as both steps share the same objective, conceptually the results from one step can be fed into another step. Such an interaction process may never converge and lead to no solution to the problem.

This paper further advances the preliminary work reported in Li et al. [19] by refining the two-step optimization approach in a case study of health care planning in rural China. Our field work indicated that residents valued distance or travel time from the nearest hospital foremost and then considered quality of care including less waiting time as a secondary desirability. Therefore, we formulate the method, termed* “two-step optimization for spatial accessibility improvement (2SO4SAI)*,*”* to account for both properties in accessibility measures. In our model, the first step is to find the best locations to site new facilities by emphasizing accessibility as proximity to the nearest facilities with several alternative objectives under consideration. The second step adjusts the capacities of facilities for minimal inequality in accessibility, where the measure of accessibility accounts for the match ratio of supply and demand and complex spatial interaction between them. By adopting one of the objectives from the traditional location-allocation problems (e.g., *p*-median, MCLP, minimax), step 1 seeks to site facilities to improve proximity for as many residents as possible and addresses the issue of efficiency. By using the popular 2SFCA to account for availability of service among competition of residents, step 2 attempts to achieve the maximal equality through adjustment in resource allocation among newly sited hospitals. Two steps combined strike a balance of dual planning objectives of efficiency and equality. The case study illustrates how the 2SO4SAI method improves both aspects of spatial accessibility for health care access (proximity and supply-demand ratio) in rural China.

## 2. Study Area and Data Preparation

The area for our case study is Xiantao, a rural county (though named* Shi*, meaning “City” in Chinese) in the mid-south of Hubei Province, China. Xiantao is in the heartland of Jiang-Han Plain, that is, the alluvial plain made by the Chang-Jiang (Yangtze) River and its largest branch Han-Shui, and has a flat topography with an area size of 2,538 km^2^. As the study focuses on spatial accessibility of health care, this section outlines data definition for three critical components: the demand side (residents), the supply side (hospitals), and the transportation linkage between them.

**Figure 1:**
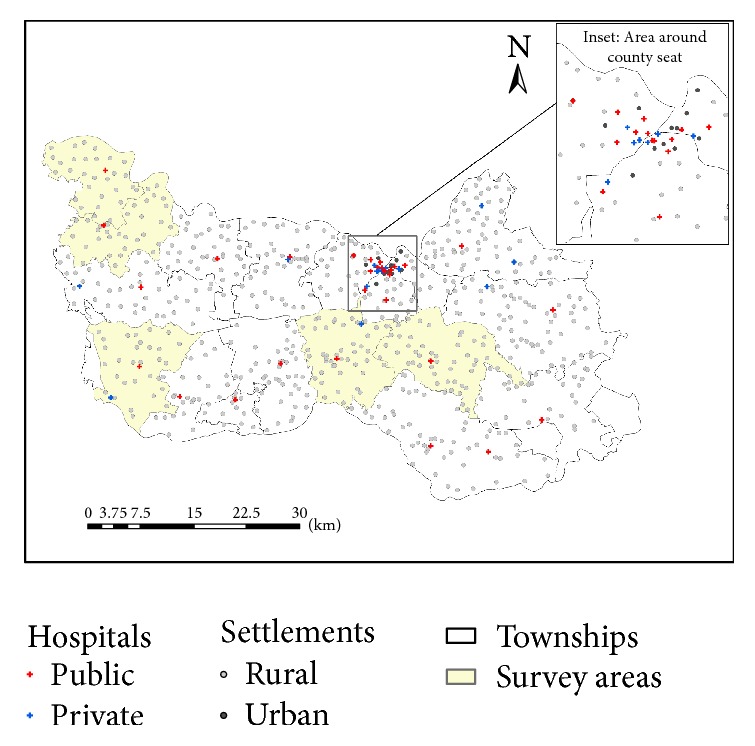
Village-level settlements and hospitals in Xiantao.

According to the 2010 census, its total population is about 1.18 million. As shown in Figure 1, all urban settlements with a population of 114,500 (14% of total population) are concentrated around the county seat in the mid-north of the study area and scattered across 12* juweihui* (the smallest administrative unit in urban China, termed “*cun* (village)” in a rural area) in three* jiedaoban* (administrative unit above juweihui in urban China, termed “zhen (township)” in rural). In addition, there are 635 villages in 18 townships in rural areas. This does not include the four water bodies in Figure 1 with no population. In sum, there are a total of 647 village-level settlements, which define the demand side of our accessibility analysis.

On the supply side, there are 44 hospitals. As shown in Figure 1, most of the hospitals (particularly the large and more specialized ones) are clustered in the urban area around the county seat, and there is at least one hospital in each township. A recent paper reports that private hospitals in China tend to concentrate in better-developed areas as they are profit driven [20]. This is not the case in our study area. Among the 14 private hospitals, 7 are in the central better-developed urban area and 7 are scattered in surrounding rural areas. Among the 30 public hospitals, 9 are administered directly by the county and the rest by townships. As summarized in Table 1, the county-level public hospitals are generally the largest in terms of bed size, doctors, and registered nurses, then the township-level public hospitals are second, and the private hospitals are the smallest. This echoes the finding from a recent study [20] that the development of private hospitals into the health care market, as a result of the health care reform launched by the Chinese government in 2009, remains on an early stage and plays a supplementary role in the overall health care market dominated by the public hospitals. Note that most hospitals in China, as it is the case in this study, provide a wide spectrum of medical care ranging from primary care to surgeries and in-patient care. The top five hospitals, all of which are county-level hospitals in the urban county seat area with more than 200 beds, provide more specialized hospital cares than the rest and may be considered as hospitals of a higher level. For the purpose of this study, we emphasize resource planning for general hospital care and do not differentiate their functions in a hierarchical sense. Our study does not cover small village-level clinics that often only have one medical practitioner on staff and no beds.

**Figure 2:**
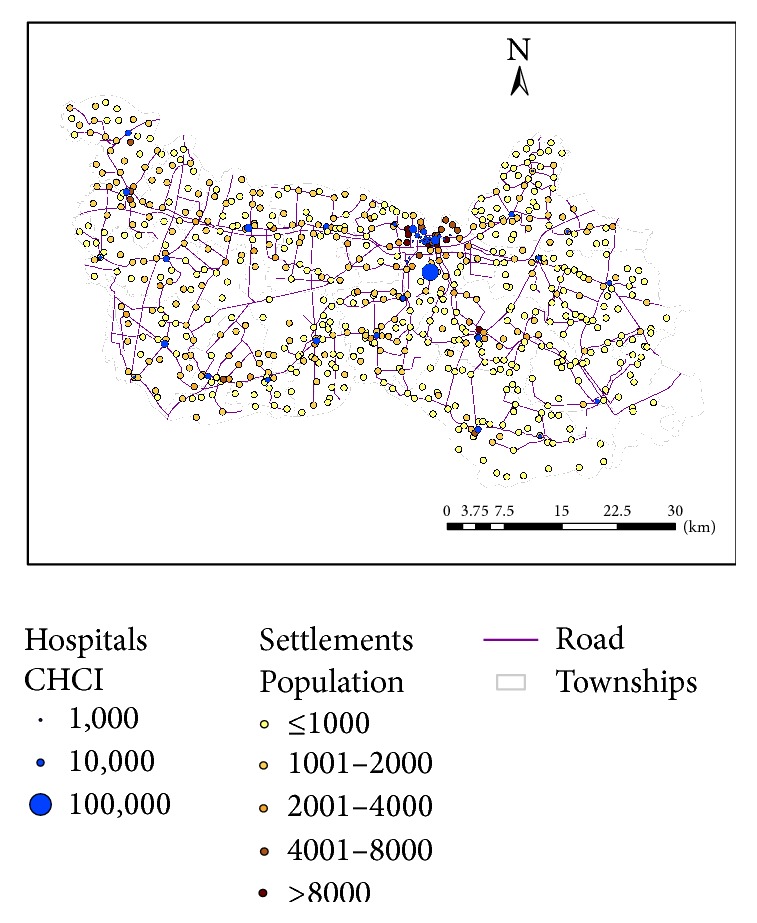
Hospital capacity, village population, and road network in Xiantao.

In order to develop a comprehensive measure for hospital capacity, we collected 14 variables including staff (licensed physicians, licensed assistant physicians, registered nurses, pharmacists, lab technicians, image technicians, other medical technicians, and administrators), medical facility (floor size in m^2^, bed size, total equipment, and major equipment), and existing patient care volumes (annual patient visits, annual in-patient discharges). A factor analysis was conducted to consolidate these 14 variables into two major independent factors, which accounted for 83% of total variance. These two factors were then synthesized to a singular index, termed* “comprehensive hospital capacity index (CHCI)*.*”* Specifically, the factors were multiplied by their corresponding loading weights and then summed up to yield the CHCI value (Figure 2). See Luo et al. [21] for details.

The paved road network, shown in Figure 2, is used to calibrate the travel impedance between villages and hospitals. The dominant transportation mode is electric motorcycles in the countryside, which travel with a speed of 25 km/h (or 15 mph) on all paved roads in the study area. There is a small segment of interstate highway that passes through the study area and is not considered for travel for hospital visits within the county because of its high toll cost and limited access. Therefore, we do not differentiate the speed on various road segments and use the shortest-path road network distance to estimate spatial impedance between villages and hospitals.

## 3. Measuring Accessibility by Proximity and 2SFCA Methods

In the summer of 2014, the research team conducted a survey in the study area that covered five townships (shown in Figure 1). Two of the townships are south of the county seat at about 10–15 km away, and the other three are on the west edge of the county at about 30–40 km away. Survey forms were distributed to 500 residents that were randomly selected to represent, evenly between males and females, various age groups (18+) and different income groups. 438 forms were returned, and 356 were valid. Among the 356 valid respondents, the vast majority (91.4%) chose hospital cares within the county citing reasons such as travel burden and financial cost (as the public health insurance system favors within-county medical cares), and the remaining 8.6% chose hospitals outside of the county (e.g., hospitals in Wuhan, the capital city of Hubei Province, which is 105 km from Xiantao's county seat). For residents choosing hospitals within the county, the travel tolerance limit was 30 minutes (equivalent to about 12.5 km) for visiting township-level hospitals for general purpose and could be stretched to as much as 2 hours for visiting county-level hospitals for specialized care. The insignificant out-of-county hospital care behavior, particularly for general (not highly specialized) hospital services, also helps limit the so-called “edge effect” in our accessibility measures without considering hospital outlets outside of the county.* Edge effect* refers to errors as a result of neglecting interaction between residents and service providers beyond a study area, especially those near the edge (borders).

**Figure 3:**
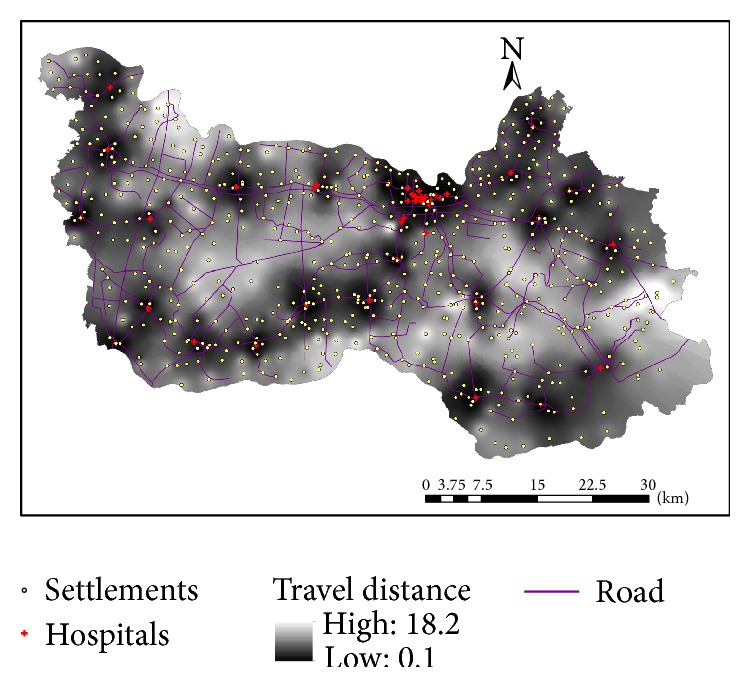
Interpolated surface for travel distance to the nearest hospital in Xiantao.

It was evident from the survey that spatial proximity was a primary concern when it came to accessibility of hospital care for rural residents in China, consistent with the finding from Li et al. [10]. This is particularly true for general cares not specialized cares, and the latter would require admission to a higher-level hospital. This research uses the travel distance from the nearest hospital as the first measure of spatial accessibility for resident at the village level. Assuming that patients choose the nearest hospital for service, such a method is also used in delineation of hospital service areas, termed “proximal area method” ([8]: 70). As shown in Figure 3, we use the interpolated surface, specifically based on the inverse distance weighted or IDW method, of travel distance to display its overall pattern, where darker color represents shorter distance and thus better accessibility. Obviously, areas closer to hospitals, especially the urban area around the county seat, enjoy better access. Based on Table 2, there is a significant urban-rural disparity: rural residents travel an average of 6.57 km to their nearest hospitals and some go as far as 18.27 km, while urban residents on average are less than 1 km away from their closest hospitals.

However, the proximity measure does not consider that residents may choose multiple hospitals, nor does it account for the competition among residents for hospital services. Based on a review of accessibility measures in the literature, Wang [14] proposed a refinement of the popular* 2-step floating catchment area (2SFCA)* method to account for both aspects. Given* m* resident locations and* n* facilities, the accessibility index is measured such as

(1) Ai=∑j=1nRjfdij

(2) Rj=Sj∑k=1mDkfdkj,

where *A*_*i*_ is the accessibility index of residents at the* i*th area, *S*_*j*_ is the supply capacity of the *j*th facility, that is, here CHCI for hospital *j*, *D*_*k*_ is the demand of the* k*th area, that is, here population in village *k*, *d*_*ij*_ or *d*_*kj*_ is the distance from residential area *i* or *k* to facility *j*,  *f* is the distance decay function, *m* is the number of residential areas, *n* is the number of facilities, *i* or *k* is the index of residential areas from 1 to *m*, and *j* is the index of facilities from 1 to *n*.

In essence, the first step in (2) assigns an initial supply-demand ratio (i.e., supply capacity divided by its surrounding residents, and the latter discounted by the distance decay effect) to each facility as a measure of its service availability, and the second step in (1) sums up these ratios for facilities around each residential area, and each ratio is also discounted by the distance decay effect. Therefore, the 2SFCA accessibility index is basically the supply-to-demand ratio, and the interaction between supply and demand is facilitated by a distance decay effect.

Note that the distance decay function *f*(*d*) may be a continuous function such as power, exponential, Gaussian, log-logistic, or a discrete function, or a hybrid between them [14]. Given the methodological emphasis of this paper, we employed the popular power function (or gravity kernel), *f*(*d*_*ij*_) = (1/*d*_*ij*_)^**β**^, and assumed the distance friction coefficient *β* = 1. The choice was made also for lack of patient flow data from residential areas to hospitals in the study area. When such data are available, one may derive the best-fitting distance decay function and its associated parameters [22, 23]. A convenient ArcGIS toolkit can be used to implement the computation of 2SFCA [24].

**Figure 4:**
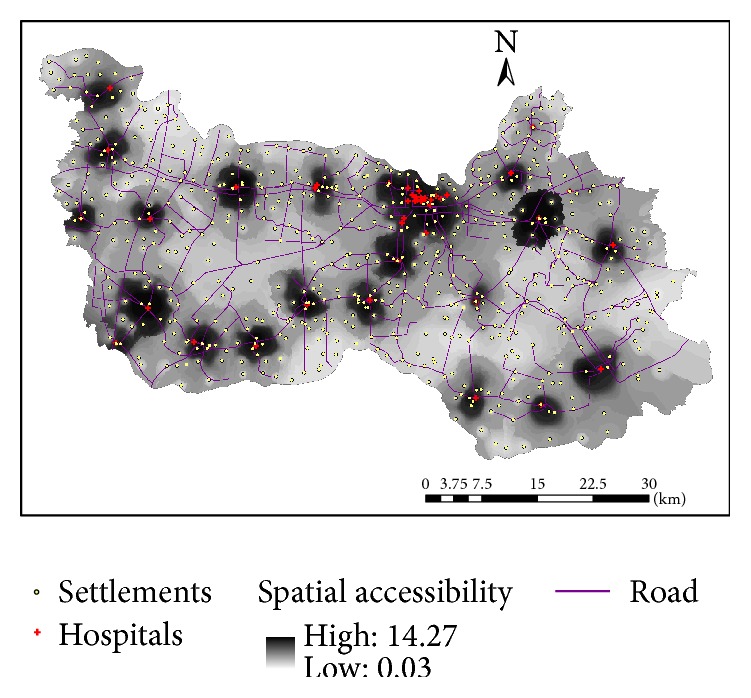
Interpolated surface for 2SFCA-based spatial accessibility to hospitals in Xiantao.

**Figure 5:**
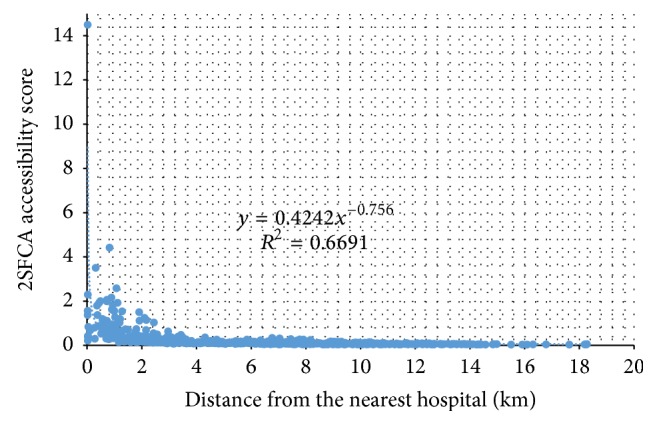
Correlation of two accessibility measures (proximity versus 2SFCA index).

The derived accessibility index value can be simply interpreted as hospital capacity CHCI per capita, and thus a higher value corresponds to better accessibility. Similarly, Figure 4 uses the IDW-interpolated surface to display its overall pattern, where darker color represents higher accessibility values and thus better accessibility. The pattern is largely consistent with Figure 3 based on proximity as areas around hospitals enjoy better accessibility, but not identical.  Figure 5 plots the two measures against each other and confirms that their correlation coefficient is as high as 0.8180 (i.e., $\sqrt{0.6691}$). However, the 2SFCA accessibility index shows a sharper contrast between areas of better and of poor access. The gradual increase in distance from the proximity method tells only one side of the story, as the 2SFCA clearly shows that those areas beyond 4 km away from their nearest hospitals have their accessibility index almost entirely below 0.2 and become below 0.1 after 6 km. Both measures reveal three pockets of poor access: the largest in an area 15–25 km southeast of the county seat, one at about the same distance range southwest of the county seat, and another small one about 5 km from the northwest corner.

The consistent patterns between the two measures echo the finding from Pan et al. 2016 and offer hope that if a planning strategy is to improve the access for these areas for one measure (e.g., reducing travel distances from hospitals), it will also improve the other measure (e.g., alleviating the stress or crowdedness in hospital services). Some recent studies on health care accessibility in China emphasize the advantages and benefits of combining the proximity and 2SFCA methods, especially when a study area is composed of both urban and rural areas [20, 25].

Based on Table 2, the urban-rural disparity in access is also revealed by the 2SFCA accessibility index: the average hospital resource per capita in urban areas (0.7961) is more than three times of that in rural areas (0.2477). Such a gap is a result of health care financing inequality in China and calls for major adjustment for policy makers to shift the investment focus toward rural and poor residents [26].

## 4. Location Optimization for Site Selection

As stated previously, we propose a method, termed* “two-step optimization for spatial accessibility improvement (2SO4SAI)*,*”* to improve accessibility for hospital care in our study area in both measures. This section discusses the first step to find the best locations to site new facilities by emphasizing accessibility as proximity to the nearest facilities.

**Figure 6:**
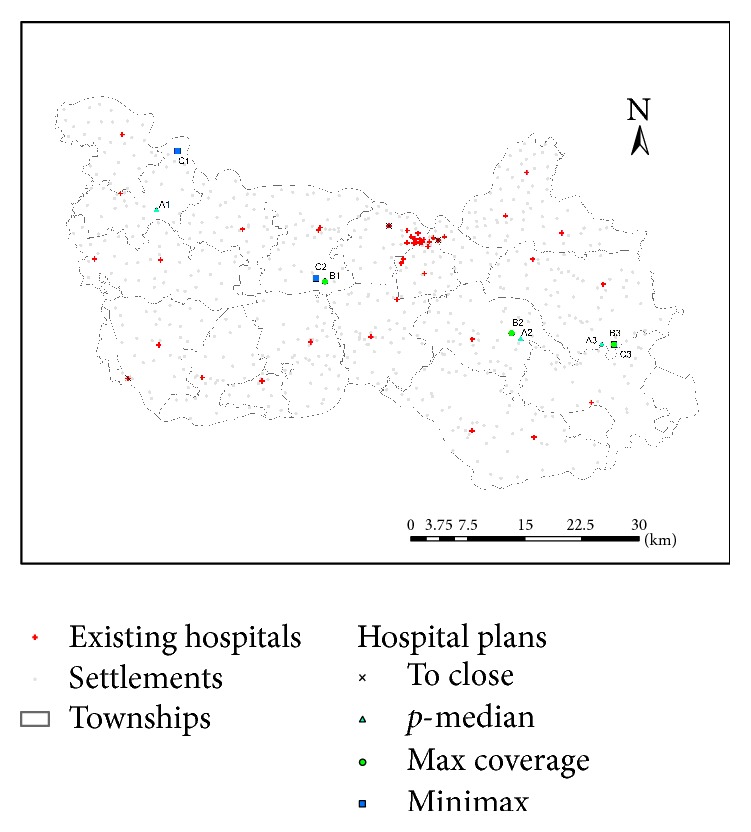
Planning scenarios for hospital site selection in Xiantao.

In 2014, the county's Bureau of Health Administration commissioned the “2020–2030 Xiantao Regional Health Statistical Plan.” One of recommendations of the plan is to close three hospitals for various reasons such as duplication of service coverage, market saturation, and subpar performance evaluation. As shown in Figure 6, two of the three hospitals are in the county seat area and the other one is at the southwest corner of the county. This research is part of the follow-up study to propose planning scenarios of replacing with three new hospitals. As discussed previously, site selection for the three hospitals is the first step in this sequential decision-making process. As revealed in the survey, travel distance is valued by residents foremost in access to hospital care. In addition, our interviews with local government officials also expressed a strong desire to reduce the travel time for residents in remote villages, especially for improving the response time for medical emergency. This leads us to seek solutions from three classic location-allocation models: the *p*-median problem, the location set covering problem (LSCP), and the minimax problem.

In our case, the *p*-median problem is to locate a given number of facilities among a set of candidate facility sites (say, among the 647 villages) so that the total travel distance from the villages to their nearest facilities is minimized. The *p*-median model formulation is

(3) Minimize: Z=∑∑DidijxijSubject to: xij≤xjj∀i,j,i≠j  each village assignment is restricted to what has been located ∑j=1mxij=1∀i  each village must be assigned to a facility ∑j=1mxjj=p∀j  exactly  p  facilities are located xij=1,0∀i,j  a village is either assigned or not to a facility,

where *i* indexes villages (*i* = 1,2,…, *n*), *j* indexes candidate facility sites (*j* = 1,2,…, *m*), *p* is the number of facilities to be located, *D*_*i*_ is the number of residents at village *i*, *d*_*ij*_ is the distance between village *i* and facility *j*, *x*_*ij*_ is 1 if village *i* is assigned to facility *j* or 0 otherwise.

Note that *p* = 44 is the total number of hospitals, and *x*_*ij*_ is preset as 1 for *j* = 1,2,…, 41 (i.e., the 41 existing hospitals that will remain open) and *x*_*ij*_ is a 0-1 binary variable to be solved for *j* = 42, 43, and 44 (i.e., the three new hospitals to be allocated). This also applies to the MCLP and minimax planning problems.

The second is the maximum covering location problem (MCLP). Here it maximizes the number of residents covered within a desired distance threshold (i.e., 10 km in our case as 87.5% of population can be covered by the existing hospitals, shown in Table 3) by locating *p* facilities and is formulated as 
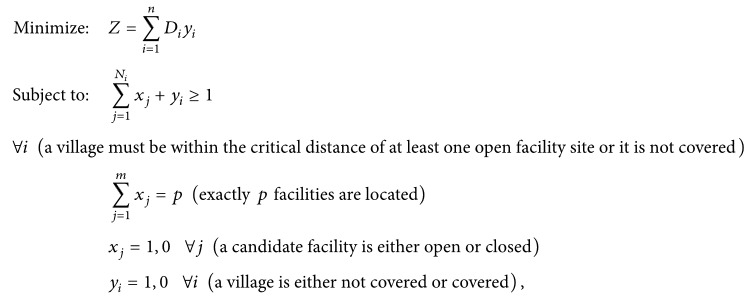
(4)where *i*, *j*, *m*, *n*, *p*, and *D*_*i*_ are the same as in the *p*-median model formulation and *N*_*i*_ is the set of facilities where the distance between village *i* and facility *j* is less than the critical distance or time *d*_0_; that is, *d*_*ij*_ ≤ *d*_0_.

Note *y*_*i*_ is 1 if a demand area *i* is not covered or 0 otherwise; thus the objective function is structured to minimize the amount of demand not covered, equivalent to maximizing the amount covered.

The third is the minimax problem. Here it minimizes the maximum distance between villages and facilities and is formulated as

(5) Minimize: Z=max⁡di ∣ 1<i<n,

where *n* is the number of villages, *d*_*i*_ is the distance between village *i* and the nearest facility to it, and max is a function to find the maximum value of a set.

To solve the three problems, given the small number (3) of hospitals to be allocated, we enumerated all possible combinations of three new hospitals out of 647 villages; that is, **C**_3_^647^ = 4,4930,915. We calculated the objective function value for each combination and found the optimal combination.

The results for the three planning scenarios are shown in Figure 6. There are overlaps and/or convergences for the optimal solutions, all of which fall in the three pockets of poor access areas. In the largest pocket in the southeast region, two of the *p*-median solutions are very close (1.5 km) to two of the MCLP ones (*A*2 and *B*2, *A*3 and *B*3), and one of the minimax solutions is even identical to that of the MCLP (*C*3 and *B*3). In the second pocket southwest of the county seat, a MCLP solution is next to a minimax one (*B*1 and *C*2). In the third pocket toward the northwest corner, a *p*-median solution and a minimax solution (*A*1 and *C*1) are about 8 km apart from each other.

As the three optimization problems focus on distance, Table 3 summarizes the results in comparison to the existing condition. All the three planning scenarios improve the access over the current state by all accounts: covering more villages and population in various distance ranges and reducing the mean and maximum distances from the nearest hospitals. Among the three scenarios, the *p*-median solution outperforms the others in the short-range (5 km) coverage, the MCLP wins in the middle-range coverage (10 km), and the minimax is the best model in the long-range coverage (15 km). As the minimax is to minimize the distance for the most remote village, its solution indeed yields the shortest maximum distance. The *p*-median yields the best solution in the mean distance to fulfill its optimization objective.

There are at least three lessons to be learned from the results:

1. All three classic location-allocation models seek to site facilities to alleviate poor accessibility in remote rural areas and thus yield some overlapping results.
2. There is some comparative advantage for each model as they have different objectives. Clearly the *p*-median model is best for generating the shortest mean distance, and the minimax is best for yielding the shortest maximum distance. The MCLP strikes a middle-ground result if one seeks a balance between the two (overall reduction in total distance versus maximum distance saving for the most distant clients).
3. In our case, the *p*-median, MCLP, and minimax models enjoy the advantage of coverage in the short, middle, and long range, respectively. Such a result is likely but not necessarily always to be applicable to other studies.

The three scenarios seem to suggest possibly five areas for new hospital sites while there are only resources for three. Following the degree of overlapping/convergence by the three models, we recommend an order of priority such as *A*3/*B*3/*C*3 > *C*2/*B*1 > *A*2/*B*2 > *A*1 > *C*1. The area of *C*2/*B*1 is placed ahead of *A*2/*B*2 as it is much farther away from the first site around *A*3/*B*3/*C*3 and may influence a completely separate region. *A*1 is ranked ahead of *C*1 as it is more inland than *C*1 on the border and thus potentially affects more surrounding villages. If we choose the first three as mandated, there will be the three clusters *A*3/*B*3/*C*3, *C*2/*B*1, and *A*2/*B*2. Given the closeness of sites within each cluster, the MCLP solution (*B*3, *B*1, and *B*2) fits the bill and makes the most sense.

In summary, by relocating three hospitals, the recommended MCLP planning scenario will cut the average distance between village residents and their nearest hospitals from 6.5 km to 6.1 km, a 6.2% overall saving, and improve the coverage at various distance ranges.

## 5. Capacity Optimization for Hospitals at the Selected Sites

As explained previously, many allocation decisions are made sequentially by first deciding the sites and then determining the capacities of selected sites. As recommended above, the three hospital sites are selected by the MCLP (*B*1, *B*2, and *B*3 in Figure 6). This section discusses the second step on how to plan for their capacities to max out their benefits. Since the previous measure of accessibility by proximity does not consider hospital capacity, we use the 2SFCA accessibility measure here to account for the intensity of competition for service (i.e., hospital capacity per capita), which subsequently affects quality of care and also outcome (e.g., [27, 28]). In addition to the rationale outlined in the Introduction (e.g., dual goals of efficiency and equality, an obligation for its source of funding from the public), there are at least two more reasons to use an equity principle to guide the design of this planning problem for resource allocation. From a practical viewpoint, a hospital in a more needy area (e.g., areas of poorer accessibility according to the 2SFCA method) needs to be provided with more resource so that it would not be too crowded to defeat its very purpose of serving. On the other side, residents in a poorer access area are likely to have poorer health, and investment of resource in the area is likely to improve their health more effectively and harness better payoff.

Here the planning problem is to find the optimal values of *S*_*j*_ for the three sited facilities (in our case, *j* = 42,43,44 to index the three new hospitals, following the 41 existing hospitals after eliminating the three recommended to be closed) so that the disparity in accessibility across villages is minimized.

Note that one important property of the 2SFCA accessibility index is that the weighted average of *A*_*i*_ (using the demand amount *D*_*i*_ as weight) equals the ratio of total supply capacities to total demand in the study area ([8]: 110-111). In other words, the mean ($\overset{-}{A}$) of accessibility is

(6) $$\begin{array}{c} \overset{-}{A}=\frac{\sum_{j=1}^{n}S_{j}}{\sum_{i=1}^{m}D_{i}}. \end{array}$$

The standard deviation ($\hat{A})$ is

(7) $$\begin{array}{c} \hat{A}=\sqrt{\frac{\sum_{i=1}^{m}{\left(A_{i}-\overset{-}{A}\right)}^{2}D_{i}}{\sum_{i=1}^{m}D_{i}}} \end{array}$$

which captures the total deviation of accessibility at each demand location weighted by the amount of demand there. The objective function is operationalized as minimizing $\hat{A}$.

In (7), ∑_*i*=1_^*m*^ *D*_*i*_ = *D* is a constant. Therefore, the objective function is equivalent to

(8) min A^⟹min⁡∑i=1mAi−A−2Di⟹min⁡∑i=1m∑j=1nSjFij−A−2Di

(9) subject to ∑j=1nSj=S,

where *S*_*j*_ is the capacity for facility *j*, *S*, *D* are the total facility capacities (CHCI) and total demand (residents), respectively, and *i*, *j*, *m*, *n*, and *D*_*i*_ are as previously defined.

In the objective function (8),  *F*_*ij*_ = *f*(*d*_*ij*_)/∑_*k*=1_^*m*^ *D*_*k*_ *f*(*d*_*kj*_) is determined since the distance matrix (its elements *d*_*ij*_ or *d*_*kj*_) and village population *D*_*k*_ are known. $\overset{-}{A}=S/D$ is also a constant. The only variable is *S*_*j*_ for the three new hospitals (*j* = 42,43,44), and other *S*_*j*_'s (*j* = 1,2,…, 41) are the capacities of existing hospitals.

In matrix notation, the objective function (8) is

(10) FS−ATDFS−DA=STFTDFS−ATDFS−STFTDA+ATDA,

where

(11) S=S1S2⋯SpTF=F11⋯F11⋮⋱⋮Fm1⋯FmpA=A−A−⋯A−T,A=mD=D1⋯0⋮⋱⋮0⋯Dm.

Dropping some constant terms, it is further transformed into a quadratic programming (QP) as follows:

(12) Minimize xTHx2+fTxsubject to Cx≤b Ex=d,

where

(13) $$\begin{array}{c} x=S\\ {H=F}^{T}DF\\ f={\left(\;-A^{T}DF\;\right)}^{T}=-F^{T}DA. \end{array}$$

**C** = −1 × **I**, and **I** is a *p* × *p* identity matrix.

(14) b=00⋯0T,b=pE=11⋯1,E=pd=S.

There are various open-source programs for solving the QP problem (http://www.numerical.rl.ac.uk/qp/qp.html). This study used Matlab, in particular its “quadprog” routine, to implement QP because of its flexibility in coding large matrices and its reliability (http://www.mathworks.com/products/matlab). See Wang and Tang [16] and Li et al. [19] for more discussion.

Here, we assume that the available resource for allocating among the three new hospitals is the total CHCI value from the three hospitals to be closed (7,237.8). In other words, our planning problem is to redistribute the resource saved from the closure of three existing hospitals to the three new hospitals. The capacity measure CHCI not only is more comprehensive than a singular measure such as number of doctors or bed size, but also offers some flexibility in the solutions for hospital administrators to reach the best combination of personnel, equipment, and facility parameters. One may interpret it as a total financial commitment.

Table 4 presents the optimization results in comparison to an arbitrary scenario, say evenly dividing the resource among the three hospitals. In terms of capacity, the planning result suggests an allocation ratio of about 1.7 : 3.0 : 1.0 for *B*1 : *B*2 : *B*3, which once again highlights the lack of hospital service in the region about 15–25 km southeast of the county seat. It calls for not only building two hospitals (*B*2 and *B*3), but also building at adequate capacities with a much larger one on the west site (*B*2). Since the total capacity of the 44 hospitals remains the same, the average accessibility index (weighted by village population) across the county is identical as 0.3911. The optimal allocation of resource (via capacity optimization) yields a slightly lower standard deviation and thus less disparity for accessibility. As stated previously, the comprehensive capacity measure, CHCI, is a linear combination of consolidated factor scores and therefore also a linear summation of the original 14 variables. The derived optimal CHCI values give the local policy makers some flexibility of various combinations of staff versus medical facility.

Using the updated capacities, a new map of 2SFCA-based accessibility was developed but not presented here as its difference from the existing pattern was visually difficult to be noted (Figure 4). The small improvement in equality is understandable since the capacity available for allocation (7,237.8) is only about 2% of the total hospital capacity in the county (317,371.8). We also experimented with different distance friction coefficients such as *β* = 1.5, 1.8, 2.0 in implementing the 2SFCA method. The allocation of capacity differed slightly from the aforementioned ratio among the three hospitals when *β* = 1, but the order of their CHCI values was consistent such as *B*2 > *B*1 > *B*3.

## 6. Conclusion

Based on the literature review, there are two popular measures of spatial accessibility: the proximity method uses the distance or travel time from the nearest facility, and the more recent 2SFCA accounts the complex spatial interaction between supply and demand and captures the availability of a service. Our field work suggests that both properties are valued by residents. A recent location-allocation model by Li et al. [19] formulates the concept of a two-step approach that first sites facilities and then determines their capacities. However, the model suffers from several technical and conceptual loopholes as stated in the Introduction and calls for refinements and more importantly a practical case study to validate it.

This paper further clarifies the sequential decision-making approach, termed “two-step optimization for spatial accessibility improvement (2SO4SAI).” The first step is location optimization but differs from the previous two-step approach in using proximity to facilities to measure accessibility and adopting the objective function from the traditional *p*-median, MCLP, or minimax problem. The second step adjusts the capacities of facilities for minimal inequality in accessibility, where the measure of accessibility is switched to 2SFCA. By adopting one of the objectives from the traditional location-allocation problems, step 1 emphasizes the efficiency principle. Step 2 strives to reduce disparity through adjustment in resource allocation among newly sited hospitals. Two steps are combined for a true hybrid optimization model that balances the dual goals of efficiency and equality. In addition, spatial proximity to facilities and a match ratio of supply and demand are two distinctive properties of accessibility. The former emphasizes the ability of reaching a service provider in the shortest time, and the latter underlines the importance of its availability as consumers compete for limited resource (captured by 2SCFA). It was our field survey that suggested the priority of proximity over availability by residents, and thus the study incorporated proximity in the first step and availability in the second step in the sequential optimization process. Simulations reported in Li et al. [19] suggest a simultaneous solution to both site selection and capacity adjustment to be very unlikely. Therefore, the sequential two-step optimization approach is both empirically justified and technically feasible.

There are several other conceivable planning scenarios: an equality objective that is based on a 2SFCA measure for accessibility and then an efficiency objective that adopts a proximity measure for accessibility, the equality objective for both steps, or the efficiency objective for both steps, and so on. Readers may develop their own case studies that truly reflect the practical challenges in a complex real world.

Our involvement in a project on planning hospitals in a rural county in central China provided us with an excellent case study to implement this method. In the case study, the planning problem is to site three new hospitals to replace soon-to-be-closed ones and make recommendation on their sizes. In this study, the spatial patterns of accessibility measured by the proximity and the 2SFCA methods are largely consistent with some minor discrepancies (e.g., more gradual change in rural areas by the proximity method than by the 2SFCA method) and reveal a significant rural-urban disparity with three pockets of poor access (all in rural areas). Based on distance from the closest hospitals, the three location optimization methods (*p*-median, MCLP, or minimax) yield results with some overlaps or convergences, and all of the selected optimal sites fall in the three pockets of poor access areas. We recommend the result by the MCLP model as its solutions overlap most with the results by the other two models and also attain a balance of desirable outcomes. The capacity optimization allocates a given amount of capacity to the three new sited hospitals. The case study demonstrated that the 2SO4SAI method improves two aspects of spatial accessibility: the location optimization reduces the average travel distance to hospitals, and the capacity optimization narrows the disparity gap in accessibility that captures hospital resource per capita.

There are some limitations for this study. As mentioned briefly in the paper, patients' behaviors in seeking hospital care are more diverse and complex than we modeled. For instance, people often bypass choices near their home for one of better reputation (e.g., a county-level instead of a township-level hospital in our case). We will need to collect and analyze the patient flow data to better model their behaviors. Such an analysis will also help us design better measures of accessibility (e.g., the choice of distance decay function and its associated parameters). Conceivably a decision problem may follow the same sequential process of siting the facility locations and then deliberating their sizes as our model but need to construct a different objective function for either step. The balance of efficiency and equality may also be approached sequentially as in this research or concurrently by a biobjective model. Even if the issue of interest remains on spatial accessibility, the specific definitions or measures may differ from the ones adopted for our study. Any combination of these extensions or adaptions will lead to a different formulation of the optimization problem and help enrich and advance the state of location-allocation analysis.

## Figures and Tables

**Table 1:** Beds, doctors, and nurses in various hospitals.

Hospital type (number)	Size	Minimum	Maximum	Mean	Standard deviation
Public: County-level (*N* = 9)	Beds	10	1040	276	329
	Doctors	5	361	78	117
	Nurses	10	601	118	190

Public: Township-level (*N* = 21)	Beds	20	158	71	29
	Doctors	6	38	15	8
	Nurses	5	49	24	10

Private (*N* = 14)	Beds	13	120	52	35
	Doctors	2	30	10	9
	Nurses	2	36	15	9

**Table 2:** Accessibility measures in rural and urban areas.

Measure	Urbanicity	Number of village-level units	Total population	Minimum	Maximum	Mean	Standard deviation
Distance from the nearest hospital (km)	Rural	635	696,931	0.02	18.27	6.57	3.91
	Urban	12	114,515	0.02	2.15	0.85	0.69
	All	647	811,446	0.02	18.27	6.47	3.95

2SFCA accessibility index	Rural	635	696,931	0.0289	14.4999	0.2477	0.6907
	Urban	12	114,515	0.1968	1.5583	0.7961	0.4277
	All	647	811,446	0.0289	14.4999	0.2579	0.6900

**Table 3:** Results of planning scenarios for hospital site selection in Xiantao.

	Number of villages			Population			Max distance (km)	Mean distance (km)
	≤5 km (%)	≤10 km (%)	≤15 km (%)	≤5 km (%)	≤10 km (%)	≤15 km (%)		
Existing	258 (39.9)	523 (80.8)	636 (98.3)	453,984 (56.0)	710,373 (87.5)	802,052 (98.8)	18.3	6.5
*p*-median	**276 (42.7)**	552 (85.3)	642 (99.2)	**472,262 (58.2)**	731,384 (90.1)	807,080 (99.5)	18.3	**6.0**
MCLP	270 (41.7)	**557 (86.1)**	641 (99.1)	464,771 (57.3)	**738,270 (91.0)**	807,834 (99.6)	17.6	6.1
Minimax	263 (40.7)	548 (84.7)	**643 (99.4)**	455,836 (56.2)	730,910 (90.1)	**809,422 (99.8)**	**16.1**	6.2

*Note*. The best solution is highlighted in *bold*.

**Table 4:** Result of capacity optimization by quadratic programming (QP).

	Resource evenly allocated scenario	Resource optimally allocated scenario
Total allocated capacity	7,237.8	7,237.8
Hospital B1 capacity^*∗*^	2,412.6	2,194.3
Hospital B2 capacity^*∗*^	2,412.6	3,760.3
Hospital B3 capacity^*∗*^	2,412.6	1,283.2
Mean of accessibility index	0.3911	0.3911
Standard deviation of accessibility index	0.5695	0.5656

*Note*. ^*∗*^See locations in Figure 6.
